# Durable remission of acute myeloid leukemia in an elderly patient following a limited course of azacitidine and venetoclax

**DOI:** 10.1016/j.lrr.2022.100345

**Published:** 2022-08-17

**Authors:** Hamid Ehsan, Qamar Iqbal, Adeel Masood, Michael R. Grunwald

**Affiliations:** a Hematology/Oncology Fellow, Levine Cancer Institute/Atrium Health, Charlotte, NC, USA; b Internal Medicine – Tidal Health Peninsula Regional. 100 East Carroll Street, Salisbury, MD 21801, USA; c Graduate Student, Master of Public Health in Epidemiology at the University of Alabama at Birmingham, AL, USA; d Leukemia Division, Department of Hematologic Oncology and Blood Disorders, Atrium Health, Levine Cancer Institute, Charlotte, NC, USA

**Keywords:** Acute myeloid leukemia (AML), Venetoclax, Azacitidine, Elderly AML, Transplant ineligible aml, Relapsed/refractory AML, Secondary AML

## Abstract

Acute myeloid leukemia (AML) is a heterogeneous hematological malignancy characterized by clonal expansion of myeloid blasts. It is the most common type of acute leukemia in adults, including elderly patients, and has historically been associated with poor outcomes in this age group. Here, we present the case of an 80-year-old woman with newly diagnosed AML with myelodysplasia-related changes. She was treated with a total of five cycles of azacitidine, two cycles as monotherapy followed by three cycles in combination with venetoclax. Therapy was stopped due to cytopenias and declining performance status. Bone marrow aspirate and biopsy immediately following treatment and again approximately four months later did not show any morphologic, immunophenotypic, or cytogenetic evidence of leukemia. The patient's clinical and performance status improved significantly with time. Follow-up labs more than three years following the completion of treatment reveal continued hematologic remission. A short treatment course of azacitidine and venetoclax with close monitoring may lead to durable responses in some patients. Further studies are necessary to determine which patients might be appropriate for treatment suspension or discontinuation while in remission.

## 1 Introduction

Acute myeloid leukemia (AML) is a heterogeneous hematologic malignancy characterized by the clonal expansion of myeloid blasts that accounts for approximately 25% of all leukemias diagnosed in adults [1]. The median age of AML diagnosis is 68 years, and patients age 65 years and older account for more than 50% of newly diagnosed AML cases [2]. The 5-year survival rate for AML is 29.5%, and historically survival for patients 65 and older has been <5% [3]. Due to aging of the population and increasing life expectancy, it is expected that the incidence of AML will continue to rise. Age is considered an independent prognostic factor for patients with AML [4]. With increasing age, there is a rise in unfavorable cytogenetic abnormalities and resistance to standard therapy [5]. Among patients younger than 56, approximately 33% are noted to have multidrug resistance compared to 57% in patients older than 75 [5]. Finally, the high prevalence of associated comorbid conditions in older patients presents a challenge.

Hypomethylating agents (HMAs) have been widely used in the treatment of elderly AML patients for well over a decade [6,7]. More recently, the combination of the HMA azacitidine with the oral B-cell lymphoma 2 (BCL2) inhibitor venetoclax has demonstrated a survival benefit compared to azacitidine plus placebo [8]. Here we present a case of an elderly AML patient treated with a fixed duration of azacitidine and venetoclax.

## 2 Case

### 2.1 Patient information

**Fig. 1:**
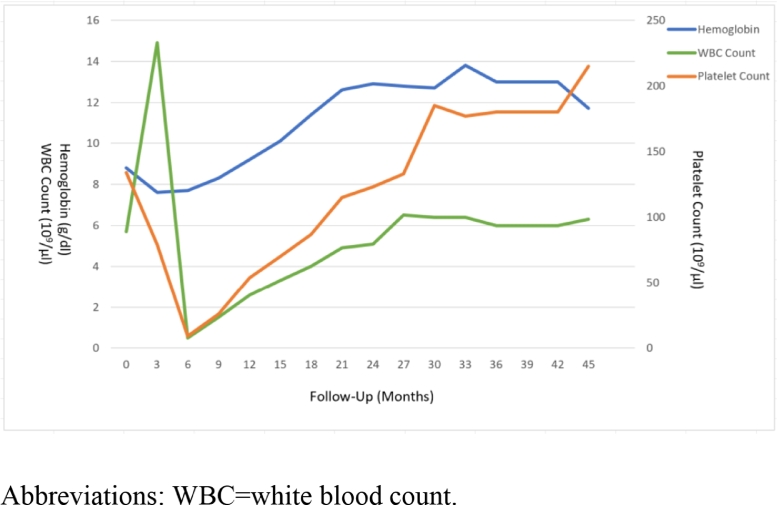
Trend in blood counts after therapy initiation. Abbreviations: WBC = white blood count.

An 80-year-old female with a past medical history significant for hyperlipidemia, coronary artery disease, and mitral valve prolapse was found to have worsening anemia and thrombocytopenia at an annual care visit. The patient's hemoglobin was noted to be 9.7 g/dL (decreased from 12.5 g/dL the previous year), white blood cell count 3.5 10^3^/ul, and platelet count 119 10^9^/ul (from 248 10^9^/ul). The peripheral blood showed atypical lymphocytes (rare forms with features suggestive of immaturity) without any distinct blast cell population and revealed occasional hypogranular granulocytes, thrombocytopenia with large platelets, and macrocytic anemia. Bone marrow biopsy and aspirate revealed AML with myelodysplasia-related changes. Karyotype was normal. Pathogenetic mutations were noted in DNMT3A (variant allele frequency [VAF] 52%), NPM1(45%), NRAS (28%), FLT3-TKD (4.4%), FLT3-ITD (1.1%), WT1(33%), and GATA2 (not reported). Karnofsky performance status was 80 at diagnosis. Given the patient's age, perceived frailty, preference, and disease status, she was initiated on azacitidine 75 mg/m^2^/day intravenously as monotherapy on days 1 to 7 of a 28-day cycle. Venetoclax 100 mg daily continuously (days 1 to 28 of a 28 day cycle) was added with cycle 3. The patient was initiated on posaconazole during cycle 4 for fungal prophylaxis. The patient completed three cycles of azacitidine and venetoclax in combination. The venetoclax dose for all cycles was 100 mg daily. The patient could not continue further therapy due to severe cytopenias and worsening performance status. She developed intracranial hemorrhage approximately six months following cycle 5, in the setting of likely underlying cerebral amyloid angiopathy and a platelet count of 32 10^9^/ul. The patient ultimately exhibited a complete neurologic recovery. Bone marrow aspirate and biopsy immediately following cycle 5 of azacitidine (cycle 3 with venetoclax) revealed a markedly hypocellular marrow. There was no morphologic or immunophenotypic evidence of acute leukemia. Myeloid molecular panel was positive for the DNMT3A (VAF 10%) mutation and negative for all other abnormalities noted at diagnosis. Four months later, bone marrow aspirate and biopsy showed morphologically normocellular trilineage hematopoiesis with adequate megakaryocytes. There was no evidence of MDS or AML. There was a continued presence of the mutation in the DNMT3A gene (VAF 19%). The patient remained in immunophenotypic and cytogenetic remission. Labs performed at nine months follow-up were significant for WBC count recovery to 1.5 10^9^/ul, while the platelet count gradually recovered to 115 10^9^/ul after 21 months. The patient's hematologic remission has now extended more than three years after completion of therapy. The timeline of the patient's related cytopenias and cell count recovery is shown in Fig. 1.

## 3 Discussion

Azacitidine and venetoclax is a generally well-tolerated regimen in newly diagnosed elderly or unfit adult AML patients. Our patient has been in complete morphologic and immunophenotypic remission after five cycles of azacitidine, the last three of which were in combination with venetoclax. The VIALE-A study was a double-blind, placebo-control phase 3 study that randomized 431 previously untreated patients ineligible for intensive induction therapy to receive azacitidine with venetoclax or azacitidine with placebo. Median age was 76 years (range: 49–91 years). Patients on the venetoclax arm (*n* = 286) received a combination of azacitidine at 75 mg/m^2^ on days 1 to 7 with venetoclax at 400 mg daily. Patients on the placebo arm (*n* = 145) received azacitidine at the same dose. Median overall survival was 14.7 months with the venetoclax regimen compared to 9.6 months in the placebo arm with a median follow-up of more than 20 months. Composite complete remission rates were 66.4% and 28.3%, respectively, with median durations of response of 17.5 and 13.4 months. Patients on the venetoclax arm received a median of 7.0 cycles of therapy; patients on the placebo arm received a median of 4.5 cycles. The most common grade III/IV hematological adverse events (AEs) with the venetoclax-containing regimen were thrombocytopenia (45%), neutropenia (42%), anemia (26%), and leukopenia (21%) [8].

It is not clear whether it is generally safe to consider elective cessation of azacitidine and venetoclax after initial response. Though azacitidine and venetoclax in combination are associated with hematological toxicity, many patients tolerate this regimen well with supportive management [9]. Recently, a retrospective analysis included 28 patients treated with combination of venetoclax with either azacitidine or low dose cytarabine (LDAC). Outcomes for 14 patients who electively stopped therapy in first remission were compared with outcomes for 14 patients who continued therapy until relapse. The main reasons to stop therapy were patient request (43%) or medical reason (57%). The median time on therapy and number of cycles were lower in the discontinuation group compared to the continuation cohort (19 vs. 32.7 months and 17 vs. 30 cycles). The patients who stopped therapy exhibited median treatment-free remission (TFR) of 46 months, with some remissions ongoing. In the discontinuation cohort, 7 (50%) patients relapsed as compared to 9 (64%) in the continuation cohort. By landmark analysis, there was no significant difference noted in progression-free survival, overall survival, or median time from relapse to death between the groups. It is noted that patients who had disease relapse were likely to exhibit clonal evolution, as evidenced by the acquisition of new cytogenetic abnormalities. Patients with NPM1 and/or IDH2 mutations seemed to do particularly well with treatment discontinuation [10].

Our patient had an NPM1 mutation at diagnosis with a VAF of 45% and a DNMT3A mutation that has persisted during morphologic and immunophenotypic remission. DNMT3A mutations can be seen in patients with disease remission, despite the loss of all other molecular AML markers [11]. In a targeted sequencing study of 482 patients with newly diagnosed AML, it was noted that persistence of DTA (DNMT3A, TET2, and/or ASXL1) mutations after therapy is not associated with increased disease relapse [12]. Our patient's case may be instructive insofar as she achieved long-term remission and full count recovery with a very limited course of combination regimen therapy and with a persistent DNMT3A mutation.

## 4 Conclusion

The azacitidine and venetoclax combination can lead to durable remission in some elderly AML patients. Our patient exhibited complete remission with therapy cessation following a period of severe cytopenias. Treatment discontinuation provides the opportunity to limit chemotherapy toxicity. This strategy requires careful further study, likely first focusing on patients experiencing toxicity and/or in those with measurable residual disease (MRD) negative complete remission.

## Authorship statement

All authors agreed to be accountable to all aspects of the work in ensuring that questions related to the accuracy or integrity of any part of the work are appropriately investigated and resolved.

## Financial disclosure statement

This manuscript is original research, has not been previously published and has not been submitted for publication elsewhere while under consideration. MRG has received consulting fees from Abbvie, Agios/Servier, Amgen, Astellas Pharma, Blueprint Medicines, Bristol Myers Squibb, Cardinal Health, CTI Biopharma, Daiichi Sankyo, Gamida Cell, Gilead Sciences, Incyte Corporation, Invitae, Karius, Novartis, Ono Pharmaceutical, Pfizer, Premier, Sierra Oncology, Stemline, and Trovagene. MRG has received institutional funds for research from Genentech/Roche, Incyte, and Janssen. HE, AM, and QI have no relevant affiliations or financial involvement with any organization or entity with a financial interest in or financial conflict with the subject matter or materials discussed in the manuscript. This includes employment, consultancies, honoraria, stock ownership or options, expert testimony, grants or patents received or pending, or royalties.

## Institutional review board statement

Not applicable.

## Informed consent statement

Obtained.

## Data availability statement

Available.

## Funding

This research received no external funding.

## CRediT authorship contribution statement

**Hamid Ehsan:** Conceptualization, Formal analysis, Writing – original draft, Writing – review & editing. **Qamar Iqbal:** Formal analysis, Writing – original draft. **Adeel Masood:** Formal analysis, Writing – original draft. **Michael R. Grunwald:** Conceptualization, Formal analysis, Writing – review & editing.

## Declaration of Competing Interest

The authors declare no conflict of interest.
